# Gubernaculum and Epididymo-Testicular Descent: Review of the Literature

**DOI:** 10.15388/Amed.2022.29.2.6

**Published:** 2022-06-29

**Authors:** Eleonora Ivanova, Beata Vincel, Gilvydas Verkauskas, Faruk Hadziselimovic

**Affiliations:** Faculty of Medicine, Vilnius University, Lithuania; Clinic of Gastroenterology, Nephrourology and Surgery, Institute of Clinical Medicine, Faculty of Medicine, Vilnius University, Vilnius, Lithuania; Clinic of Gastroenterology, Nephrourology and Surgery, Institute of Clinical Medicine, Faculty of Medicine, Vilnius University, Vilnius, Lithuania; Institute for Cryptorchidism Research, Kindermedizinisches Zentrum, Liestal, Switzerland

**Keywords:** cryptorchidism, epididymo-testicular descent, gubernaculum, Insl3

## Abstract

Cryptorchidism is a common disorder in boys that has been widely studied both experimentally and clinically. The role of the gubernaculum, a mesenchymal tissue extending from the fetal testis and epididymis to the developing scrotum, is still unclear. Even the name is debated: ‘gubernaculum epididymis’ or ‘gubernaculum testis’. This review does not aim to provide a global overview of competing theories on testicular descent, but focuses on the role of the gubernaculum in epididymo-testicular descent. We identified four major pitfalls of gubernaculum research: the role of the gubernaculum, of insulin-like peptide 3, anti-Müllerian hormone, and androgens. The major critical issues were that the gubernaculum plays a guiding role for the epididymis, descending prior to the testis and expanding the inguinal canal; insulin-like peptide 3 is not as important for the process of descent in humans as the rate of insulin-like peptide 3 mutations is low; anti-Müllerian hormone plays no significant role in epididymo-testicular descent; androgens and gonadotropins play a crucial role in epididymo-testicular descent. The role of the epididymis in the complex process of gubernaculum, epididymis, and testis migration is underestimated and should be included in future research.

## Santrauka.

Nunusileidusios sėklidės (kriptorchizmas) yra dažna vyriškų lytinių organų patologija. Sėklidės pavadis (lot. *gubernaculum testis*) – mezenchiminis audinys tarp vaisiaus sėklidės ir prielipo bei besiformuojančio kapšelio. Jo reikšmė sėklidei ir prielipui nusileisti nėra aiški. Šios apžvalgos tikslas nėra įvertinti vienų kitoms prieštaraujančių sėklidės nusileidimo teorijų, o susitelkiama ties sėklidės pavadžio (ar dar kitaip vadinamo prielipo pavadžio) svarba sėklidei nusileisti. Mes kėlėme šiuos pagrindinius klausimus apie sėklidės pavadį: sėklidės pavadžio svarba, į insuliną panašaus hormono 3, antimiulerinio hormono ir androgenų reikšmė. Sėklidės pavadis atlieka tik orientacinį vaidmenį prielipui ir leidžiasi prieš sėklidę plėsdamas kirkšnies kanalą. Į insuliną panašus hormonas nėra svarbus sėklidei nusileisti, nes žmonių populiacijos šio geno mutacijos yra retos. Antimiulerinis hormonas neatlieka reikšmingo vaidmens, kad sėklidė ir prielipas nusileistų. Androgenai ir gonadotropinai yra svarbiausi, kad įvyktų sėklidės ir prielipo nusileidimas. Prielipo reikšmė sudėtingame pavadžio, prielipo ir sėklidės migracijos procese yra nepakankamai įvertinta.

## Introduction

**Figure 1.**
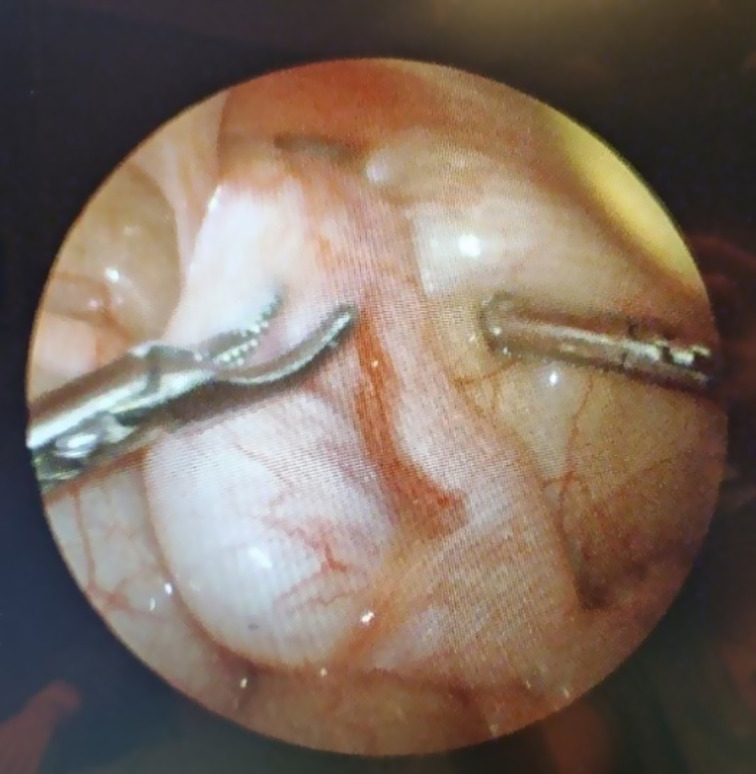
Laparascopic view of an intra-abdominal testis showing prior descent of the body and tail of the epididymis

**Figure 2.**
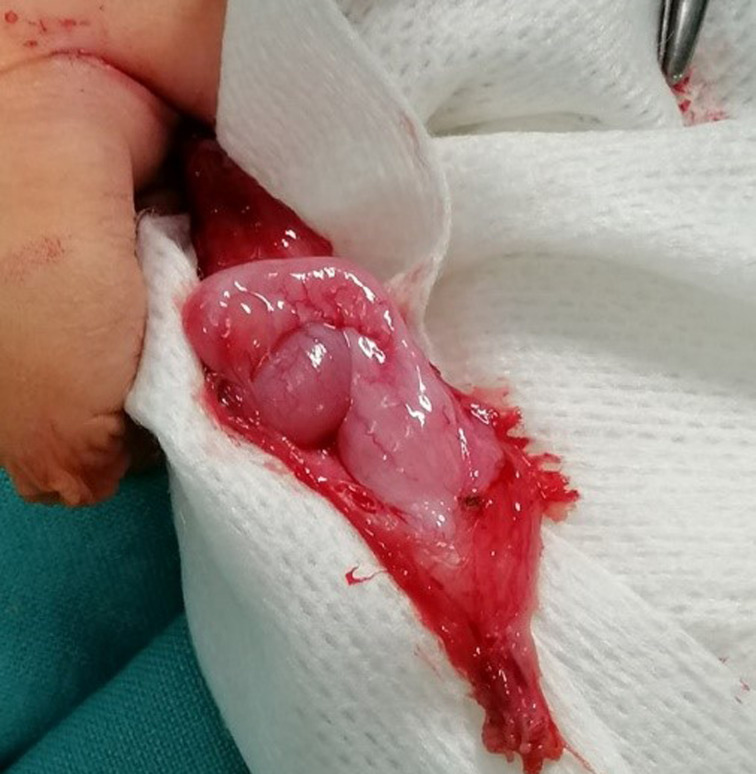
Prescrotal testis showing prior descent of the body and tail of the epididymis

Cryptorchidism, one of the most common disorders in boys, has been widely studied both experimentally and clinically. Many review articles have been published, but the main conclusions remain debatable. In trying to understand testicular descent, one of the most controversial issues is the gubernaculum. Though it may seem that this topic is all sorted out, upon a closer look, even the name ‘gubernaculum testis’ or ‘gubernaculum epididymis’ needs to be decided [1]. Of course, in the heads of many people, testis and epididymis are no more than a ‘testicle’, but for a surgeon operating on an undescended testis it is more complex (shown in Figs. 1, 2).

The gubernaculum, the mesenchymal tissue extending from the fetal epididymis and testis to the developing scrotum, was originally named by John Hunter [2]. Later, many theories emerged that assigned the gubernaculum different active and passive roles. Seiler characterized the gubernaculum as a muscle-like structure, whereas Cleland described it without such tissue [3,4]. However, the gubernaculum was given an active role of pulling the testis down to the scrotum by both investigators. The passive role of gubernaculum was advocated by Weil’s intra-abdominal pressure hypothesis [5].

Gubernaculum research is mostly conducted in rodents. The rodent gubernaculum differs from human gubernaculum in terms of structure, migration time, and last step of descent. A major difference is that the rodent gubernaculum is fibrous with a circumferential cremaster muscle, but the human gubernaculum is jelly mesenchymatous [6]. In addition, testicular descent in humans is complete at birth (approximately 35th week of gestation), whereas complete testicular descent in rodents occurs at puberty. Moreover, in humans, the descent ends with closure of the *processus vaginalis* and in rodents it remains open with a fat pad on the epididymis, which helps prevent inguinal hernias [7,8].

## Main Text

In this review, we discuss the four major pitfalls met earlier and still potentially misleading the research on this common congenital disorder in boys:

- The role of the gubernaculum;
- The link between hypogonadism and epididymo-testicular descent;
- The role of insulin-like peptide 3 (Insl3) in epididymo-testicular descent;
- The role of anti-Müllerian hormone (AMH) in epididymo-testicular descent.

Literature to review was found in the PubMed database excluding case reports, editorials, and opinions.

### Pitfall No. 1 – The role of the gubernaculum

The human gubernaculum consists of a fibrous structure rich in collagen and elastic fibers without contractile potential and extracellular matrix [9]. It undergoes remodeling during descent with a trend towards fibrous tissue [10]. The most important question about the function of the gubernaculum remains is whether it holds the testis near the inguinal region and migrates towards the scrotum, clearing the space ahead, or the epididymis holds the testis while descending with the gubernaculum [8,11].

In 1978, Bergh et al. reported that testis descent can occur with a severed proximal gubernaculum, whereas testicular descent is prevented when the distal gubernaculum is cut. In addition, descent of the epididymis can occur in the absence of a testis [12]. These findings highlight the importance of the epididymis in epididymo-testicular descent. However, many of the later studies continued research in which gubernaculum was given the key role, disregarding the findings by Bergh et al. For example, Favorito et al. stated that differentiation of the muscular gubernacular bulb results in traction through the inguinal region [13]. Other ideas derived from schematic (hypothetical) presentations of epididymo-testicular descent led to the conclusion that the gubernaculum retains the testis close to the inguinal canal, whereas the rest of the abdominal contents grow dorsally [14]. Even recent papers exclude the epididymis from schematic presentations [15].

Going back to the findings reported by Bergh et al. and taking a closer look at the epididymis reveals interesting discoveries. In 2012, Rachmani et al. showed that the degree of nonfusion between the testis and epididymis is associated with a higher position of the testis. Total nonfusion reliably interferes with descent [16]. In 2014, Caterino et al. reported a large series of 1002 cryptorchid patients and 230 controls, examining the surgical data for evidence of anomalies in epididymal/testicular fusion. The cryptorchid group had a significant number of epididymal/testicular fusion anomalies compared to the control group. This finding correlated with persistence of the *processus vaginalis* and more proximal location of the testis [17]. In general, epididymal anomalies are common in cryptorchid boys, and testicular-epididymal fusion anomalies are associated with intra-abdominal cryptorchidism [18,19]. An improper connection between the epididymis and testis may lead to cryptorchidism. The true morphogenesis of this junction is not yet clear, but several genes have been implicated, including paired-Box 2/8 (*Pax2/8*) *and* fibroblast growth factor [20]. In 1984, Hadziselimovic reported that August-Copenhagen-Irish cryptorchid rats lacking an epididymis had no transabdominal descent and, in 2017, presented histological sections that showed the epididymis precedes the testis throughout descent by the gelatinous gubernaculum dilating the inguinal canal [11,1]. In 1986, Heyns dissected 178 male human fetuses and found that ‘the gubernaculum, epididymis, and testis appear to move through the inguinal canal as a unit, covered on its anterior aspect by the open *processus vaginalis*’*.* Furthermore, no extension of the gubernaculum into the bottom of the scrotum exists [21,22]. In 2020, a three-dimensional (3D) histological reconstruction of testicular descent in mice showed the tail of the epididymis being pulled down to the inner ring by the gubernaculum, and then the tail and body of the epididymis expanding the inner ring and inguinal canal through their morphological changes. The epididymis then continued to go down into the scrotum and expand it. Finally, the testis and the head of the epididymis followed the body of the epididymis and entered the scrotum through the enlarged inner ring and inguinal canal. This shows that the epididymis descends prior to the testis. In regards to the role of the gubernaculum, according to the 3D model, it is not directly anchored to the testis itself and plays a guiding role for the epididymal tail and testis [23]. This supports the idea that the gubernaculum should be called ‘gubernaculum epididymis’ rather than ‘gubernaculum testis’.

Genetic studies on the etiology of cryptorchidism also support the importance of the epididymis for testicular descent. Recent research by Jorgez et al. showed that E2F transcription factor-1 (E2F1) regulates genes required for testicular descent, such as *Wnt4, Insl3, Ar, Lgr8, Hoxa10, Amh, Dmrt1*, and *Fst*. In addition to spermatogenic failure and gubernacular defects, *E2F1*-null cryptorchid mice have a significant decrease in epididymal size compared to wild-type individuals [15].

Both gubernaculum and cremaster muscle are a derivative of mesenchyme and have a distinct myosinic pattern [24]. The epididymis exhibits spontaneous contractions along the epididymal duct through cAMP-mediated smooth muscle contractions [25]. Histological investigation has also shown the presence of smooth muscle around the epididymal duct [26]. This suggests a common molecular mechanism for mesenchymal-epithelial transition involved in the process of epididymo-testicular descent.

### Pitfall No. 2 – Hypogonadism and epididymo-testicular descent

The next topic of debate is the role of hypogonadism and androgens in epididymo-testicular descent and whether it is staged. Clarnette et al. stated that, ‘in conditions of androgen insensitivity, the testis is located in the inguinal region, indicating that the first phase of descent is normal, but the inguinoscrotal descent has failed to occur’ [27]. Antiandrogen treatment with flutamide has been shown to induce cryptorchidism, epididymal anomalies, and failure of gubernacular regression in pigs, affecting both transabdominal and transinguinal descent of the testes [28]. In subjects with complete and partial androgen insensitivity syndrome, the complete female phenotype with abdominal testes was found in 86% of patients, and this incidence decreased significantly with increasing masculinization [29].

Mutations in Wilms’ tumor gene (*WT1*) may be associated with testicular maldescent and genital abnormalities consistent with defects in the androgen pathway of testis descent [30]. Studies on *WT1* have shown that androgens are important for both intra-abdominal and inguinoscrotal descent, but multiple other factors may modulate this process [31]. An inverse relationship between *androgen receptor* and *WT1* expression has been found in prostate cancer cell lines, together with WT1 repression of the androgen receptor promoter [32]. The early growth transcription factor family response to gonadotropin-releasing hormone (GnRH) treatment indicates involvement of gonadotropins, androgens, and WT1 in the process of testicular descent [33,34].

Measuring testosterone production in testes from 18-day-old fetal mice showed that active gonadotropin material is present in the pituitary gland and exerts significant actions on the production of testosterone in the fetal testis [35]. The importance of gonadotropins in the development of fertility is highlighted when investigating luteinizing hormone receptor knockout (LuRKO) mice. These mice exhibit dramatically reduced Leydig cell counts and arrested spermatogenesis [36]. Further research on LuRKO mice has shown that testosterone replacement therapy corrects the cryptorchidism. This confirms that testosterone facilitates the completion of testicular descent [37]. Migratory arrest of GnRH neurons to the hypothalamus in transgenic mice has been shown to be the cause of hypogonadotropic hypogonadism, resulting in crypto-epididymis, severe gonadal hypoplasia and, consequently, infertility [38].

Androgens and estrogens are involved in multiple processes in both males and females. Male offspring of estrogen-treated mice present with low levels of pituitary luteinizing hormone, Leydig cell atrophy, and low testicular testosterone, resulting in abnormal epididymal development and cryptorchidism [39]. Control mice clearly exhibited a sharp increase in testicular testosterone around embryonic days 17 and 18. This was completely missing in estrogen-treated cryptorchid mice [40]. When pregnant female mice were given injections of estradiol, all of their male offspring lacked gubernacula and the testes were freely mobile within the abdomen [41,42].

At least partial reversal of estrogen action by human chorionic gonadotropin administration suggested that the leading cause of epididymo-testicular maldescent is induced by an insufficiency of the hypothalamo-pituitary-gonadal axis [39]. In summary, the data provided above confirms the crucial role of androgens for epididymo-testicular descent, both abdominal and inguinoscrotal. It also supports the hypothesis of hypogonadotropic hypogonadism as the cause of cryptorchidism.

### Pitfall No. 3 – Role of Insl3 in epididymo-testicular descent

Over the last 30 years, many theories have been proposed to explain epididymo-testicular descent. The most important factor responsible for this step was considered to be Insl3, a major product of fetal and adult Leydig cells [14]. The role of Insl3 in the descent of the testes was evoked after two studies found that Insl3 knockout mice have intra-abdominal undescended testes and impaired development of the gubernacula [43,44]. Later studies in mice revealed the importance of both Insl3 and androgens for transabdominal testicular descent [45]. When LuRKO mice received testosterone replacement therapy, that had no impact on Insl3 expression but restored Insl3 receptor expression in the gubernaculum. This suggests that testosterone maintains normal receptor levels and even low INSL3 levels can activate them [37].

Analyzing pictures of Insl3-null mice, one should notice not only a gubernacular abnormality, but abnormal cauda epididymis, though this was not acknowledged in the paper [44]. In a similar study by Kubota et al., the epididymides of Insl3^-/-^ mice look abnormal [46]. Overlooking these observations may underestimate the role of the epididymis in the descent of the epididymo-testicular unit. Abnormal epididymides in Insl3-deficient mice suggest an impact of Insl3 on the development of the epididymis rather than the testis [26].

With all of the evidence on the significance of Insl3 in rodent testicular descent, the question remains: how important is the INSL3-LGR8/G-protein-coupled receptor (GREAT) for cryptorchidism in humans? When trying to replicate the motivating results, many studies were looking for INSL3 gene mutations in former cryptorchid boys and men; surprisingly, it appeared at extremely low rates. The cumulative incidence of INSL3 gene mutations in eight studies with a total of 859 patients was 2.3% (Table 1). Similar results were obtained when searching for GREAT gene mutations; the cumulative incidence from six studies with a total of 990 patients was 3.1% (shown in Table 1). It is also worth mentioning that GREAT mutations were present in patients with retractile testes or spontaneous testicular descent (i.e., not really cryptorchid). This may indicate that GREAT mutation does not have a direct effect on testicular descent in this group [47–49]. Based on the data provided in the 12 studies, INSL3/GREAT mutations are not as important in the development of cryptorchidism in humans as previously thought.

### Pitfall No. 4 – Role of anti-Müllerian hormone in epididymo-testicular descent

AMH is another factor commonly discussed in the process of testicular descent. It is produced by Sertoli cells and causes the regression of Müllerian ducts [59]. Clarnette et al. performed a literature review and came to the conclusion that, in conditions in which AMH is absent, the gubernaculum is ‘feminized’, resulting in a testis position normally occupied by an ovary [27]. Moreover, the review supported the hypothesis of the biphasic hormone-dependent model for testicular descent proposed by Hutson [60]. Based on the aforementioned review, Clarnette et al. agreed that the ‘transabdominal’ phase of testicular descent is AMH-dependent and controls the swelling reaction in the male gubernaculum [27]. However, several studies published before and after have shown that these ideas about AMH were not correct. Lyet et al. analyzed transgenic mice expressing human AMH throughout prenatal life and found that this hormone had no effect on the growth and development of the gubernaculum [61]. Another study performed on AMH-deficient mice found that they have normal epididymo-testicular descent [62]. This result was confirmed by another study on disruption of AMH receptor genes [63]. Moreover, a Emmen et al. showed that AMH has no effect on the gubernaculum [45]. Thus, it seems that AMH alone has no significant role in epididymo-testicular descent.

**Table 1.** Cumulative incidence of INSL3/GREAT gene mutations in 12 studies

Author	INSL3 incidence	GREAT incidence	Remarks
Incidence of mutations found; research groups were not uniform/homogeneous			
Ferlin et al. 2003 [47]	4.6% (4/87)	4.6% (4/87)	Study included five patients with retractile testes. One of the GREAT mutations was present in a patient with retractile testis.
Foresta et al. 2004 [48]	4.4% (6/135)	3% (4/135)	Study included eight patients with retractile testes. One of the GREAT mutations was present in a patient with retractile testis.
Bogatcheva et al. 2007 [49]	-	3.3% (20/598)	T222P mutation. Study included 94 patients with spontaneous testicular descent. One of the GREAT mutations was present in one of these patients.
El Houate et al. 2007 [50]	2.75% (3/109)	-	Study included 19 patients with cryptorchidism and hypospadias, and 1 patient with bilateral cryptorchidism and micropenis. One of the INSL3 mutations was detected in a patient with bilateral cryptorchidism and micropenis.
Incidence of mutations found; uniform research groups			
Canto et al. 2003 [51]	2.7% (4/150)	-	DNA obtained from patients with cryptorchidism. Two of the mutations did not alter the encoded amino acid and one was also found in the controls.
Tomboc et al. 2000 [52]	1.4% (2/145)	-	Patients who underwent surgical correction for cryptorchidism, no histological diagnosis.
Gorlov et al. 2002 [53]	-	1.6% (1/61)	Unilateral and bilateral cryptorchidism, no histological diagnosis.
Research groups with 0% incidence of mutations or mutations with no clinical significance			
Baker et al. 2002 [54]	0% (0/118)	-	Unilateral and bilateral cryptorchidism.
Koskimies et al. 2000 [55]	0% (0/30)	-	Seven patients had possible familial form.
Lim et al. 2001 [56]	1.2% (1/85)	-	Study included patients with undermasculinized genitalia. The only mutation was found in the patient with completely female genitalia, bilateral intra-abdominal testes.
El Houate et al. 2008 [57]	-	2.75% (3/109)	T222P mutation. No association with cryptorchidism found (incidence in control group: 4/250).
Nuti et al. 2008 [58]	-	3.6% (13/359)	T222P mutation found to not be causative. Difference with incidence in controls not significant (1.7%, 8/463).
Total	2.3% (20/859)	3.4% (45/1349)	

INSL3, insulin-like peptide 3; GREAT, G-protein-coupled receptor affecting testis descent.

## Conclusion

Most experimental evidence supports the leading role of gonadotropins and androgens in epididymo-testicular descent. However, the role of the epididymis is underestimated. Future research should not omit the role of the epididymis in the complex process of gubernaculum, epididymis, and testis migration.

## References

[1] Hadziselimovic F. On the descent of the epididymo-testicular unit, cryptorchidism, and prevention of infertility. Basic Clin Androl. 2017 Dec 1;27. 10.1186/s12610-017-0065-8 PMC5686796 29163975

[2] Hunter J. A description of the fituation of the testis in the foetus, with its decent into the scrotum. In: Observations on Certain Parts of the Animal Oeconomy. Philadelphia: Haswell, Barrington; 1786. p. 1–261.

[3] Seiler BW. Observationes nonnullae de testiculorum ex abdomine in scrotum descensu et partium genitalium anomaliis. G. Engelmann; 1817.

[4] Cleland, John. The mechanism of the gubernaculum testis : with an introductory sketch of the development of the testes, and an appendix on the purpose of their descent from the abdomen. Edinburgh; 1856.

[5] Weil C. Ueber den Descensus testiculorum: nebst Bemerkungen uebeer die Entwickelung der Scheidenhaute und des Scrotums. Leipzig; 1885.

[6] Hadziselimovic F. Embryology of testicular descent and maldescent. In: Cryptorchidism: Management and Implications. Berlin Heidelberg: Springer-Verlag; 1983. p. 10–34.

[7] Hutson JM, Baskin LS, Risbridger G, Cunha GR. The power and perils of animal models with urogenital anomalies: Handle with care. J Pediatr Urol. 2014 Aug;10(4):699–705. 24768568 10.1016/j.jpurol.2014.03.003 PMC4454504

[8] Hutson JM, Thorup JM, Beasley SW. Inguinoscrotal descent of the testis. In: Descent of the Testis. 2nd ed. Springer International Publishing; 2016. p. 29–41. 10.1016/j.jpurol.2014.03.003

[9] Costa WS, Sampaio FJB, Favorito LA, Cardoso LEM. Testicular migration: remodeling of connective tissue and muscle cells in human gubernaculum testis. J Urol. 2002 May;167(5):2171–6. 11956474

[10] Soito ICS, Favorito LA, Costa WS, Sampaio FJB, Cardoso LEM. Extracellular matrix remodeling in the human gubernaculum during fetal testicular descent and in cryptorchidic children. World J Urol. 2011 Aug;29(4):535–540. 10.1007/BF00255914 21626117

[11] Hadziselimović F. Mechanism of testicular descent. Urol Res. 1984;12(3):155–157. 10.1111/j.1365-2605.1978.tb00605.x 6148796

[12] Bergh A, Helander HF, Wahlqvist L. Studies on Factors Governing Testicular Decent in the Rat – Particularly the Role of Gubernaculum Testis. Int J Androl. 1978;1(1–6):342–356. 10.1111/j.1365-2605.1978.tb00605.x

[13] Favorito LA, Costa SF, Julio Junior HR, Sampaio FJB. The importance of the gubernaculum in testicular migration during the human fetal period. Int Braz J Urol. 2014 Dec);40(6):722–729. 10.1590/S1677-5538.IBJU.2014.06.02 25615240

[14] Ivell R, Hartung S. The molecular basis of cryptorchidism. Mol Hum Reprod. 2003 Apr 1;9(4):175–181. 10.1093/molehr/gag025 12651898

[15] Jorgez CJ, Seth A, Wilken N, Bournat JC, Chen CH, Lamb DJ. E2F1 regulates testicular descent and controls spermatogenesis by influencing WNT4 signaling. Development. 2021 Jan 13;148(1):dev191189. 10.1242/dev.191189 33441379 PMC7823160

[16] Rachmani E, Zachariou Z, Snyder H, Hadziselimovic F. Complete testis-epididymis nonfusion anomaly: a typical association with cryptorchid testis. Urol Int. 2012;89(3):355–7. 10.1159/000342665 23037312

[17] Caterino S, Lorenzon L, Cavallini M, Cavaniglia D, Ferro F. Epididymal-testicular fusion anomalies in cryptorchidism are associated with proximal location of the undescended testis and with a widely patent processus vaginalis. J Anat. 2014 Oct);225(4):473–478. 10.1111/joa.12222 25048056 PMC4174029

[18] Marshall FF, Shermeta DW. Epididymal abnormalities associated with undescended testis. J Urol. 1979 Mar);121(3):341–343. 10.1016/s0022-5347(17)56780-4 34733

[19] Qin KR, Morley C, Nataraja RM, Pacilli M. The spectrum of testicular-epididymal fusion anomalies in children with cryptorchidism: Personal experience, systematic review and meta-analysis. J Ped Urol. 2020 Apr);16(2):124–129. 10.1016/j.jpurol.2019.12.016 32008986

[20] de Mello Santos T, Hinton BT. We, the developing rete testis, efferent ducts, and Wolffian duct, all hereby agree that we need to connect. Andrology. 2019 Sep);7(5):581–587. 10.1111/andr.12631 31033257 PMC6688955

[21] Heyns CF. The gubernaculum during testicular descent in the human fetus. J Anat. 1987 Aug;153:93–112. https://www.ncbi.nlm.nih.gov/pmc/articles/PMC1261785/2892824 PMC1261785

[22] Bouzada J, Vázquez T, Duran M, Delmas V, Larkin T, Cuesta MA, et al. New insights into the morphogenesis of the gubernaculum testis and the inguinal canal. Clin Anat. 2017;30(5):599–607. 10.1002/ca.22880 28422355

[23] Wu H, Xie X, Li J. Application of three dimensional histological reconstuction technology research testicular descent on mice. Chin J Appl Clin Pediatr. 2020;35(17):1320–1324. http://rs.yiigle.com/CN101070202017/1238793.htm

[24] Molinaro F, Fusi G, Aglianò M, Volpi N, Franci D, Lorenzoni P, et al. Clinically Differentiated Abnormalities of the Architecture and Expression of Myosin Isoforms of the Human Cremaster Muscle in Cryptorchidism and Retractile Testis. Urol Int. 2020;104(11–12):891–901. 10.1159/000508432 32674099

[25] Mietens A, Tasch S, Stammler A, Konrad L, Feuerstacke C, Middendorff R. Time-lapse imaging as a tool to investigate contractility of the epididymal duct--effects of cGMP signaling. PloS One. 2014;9(3):e92603. 10.1371/journal.pone.0092603 24662987 PMC3963912

[26] Hadziselimovic F, Adham I. Insulin 3-like hormone and its role in epididymo-testicular descent. Int Braz J Urol. 2007 Jun);33(3):407–411; discussion 411-413. 10.1590/s1677-55382007000300015 17626659

[27] Clarnette TD, Sugita Y, Hutson JM. Genital anomalies in human and animal models reveal the mechanisms and hormones governing testicular descent. Br J Urol. 1997 Jan 1;79(1):99–112. 10.1590/s1677-55382007000300015 9043507

[28] McMahon DR, Kramer SA, Husmann DA. Antiandrogen induced cryptorchidism in the pig is associated with failed gubernacular regression and epididymal malformations. J Urol. 1995 Aug;154(2 Pt 1):553–557. 10.1097/00005392-199508000-00068 7609135

[29] Barthold JS, Kumasi-rivers K, Upadhyay J, Shekarriz B, Imperato-mcginley J. Testicular position in the androgen insensitivity syndrome: implications for the role of androgens in testicular descent. J Urol. 2000 Aug 1;164(2):497–501. 10.1016/S0022-5347(05)67411-3 10893634

[30] Köhler B, Delezoide AL, Boizet-Bonhoure B, McPhaul MJ, Sultan C, Lumbroso S. Coexpression of Wilms’ tumor suppressor 1 (WT1) and androgen receptor (AR) in the genital tract of human male embryos and regulation of AR promoter activity by WT1. J Mol Endocrinol. 2007 May 1;38(5):547–554. 10.1677/JME-06-0020 17496156

[31] Lim HN, Hughes IA, Ross Hawkins J. Clinical and molecular evidence for the role of androgens and WT1 in testis descent. Mol Cell Endocrinol. 2001 Dec 20;185(1):43–50. 10.1016/S0303-7207(01)00631-1 11738793

[32] Zaia A, Fraizer GC, Piantanelli L, Saunders GF. Transcriptional regulation of the androgen signaling pathway by the Wilms’ tumor suppressor gene WT1. Anticancer Res. 2001 Feb;21(1A):1–10. 11299720

[33] Hadziselimovic F, Gegenschatz-Schmid K, Verkauskas G, Demougin P, Bilius V, Dasevicius D, et al. GnRHa Treatment of Cryptorchid Boys Affects Genes Involved in Hormonal Control of the HPG Axis and Fertility. Sex Dev. 2017;11(3):126–36. 10.1159/000471937 28505621

[34] Ritchie MF, Yue C, Zhou Y, Houghton PJ, Soboloff J. Wilms Tumor Suppressor 1 (WT1) and Early Growth Response 1 (EGR1) Are Regulators of STIM1 Expression. J Biol Chem. 2010 Apr 2;285(14):10591–10596. 10.1074/jbc.M109.083493 20123987 PMC2856267

[35] Pointis G, Mahoudeau JA. Demonstration of a pituitary gonadotrophin hormone activity in the male foetal mouse. Acta Endocrinol. 1976 Sep);83(1):158–65. 10.1530/acta.0.0830158 183442

[36] Zhang FP, Poutanen M, Wilbertz J, Huhtaniemi I. Normal Prenatal but Arrested Postnatal Sexual Development of Luteinizing Hormone Receptor Knockout (LuRKO) Mice. Mol Endocrinol. 2001 Jan 1;15(1):172–183. 10.1210/mend.15.1.0582 11145748

[37] Yuan FP, Lin DX, Rao CV, Lei ZM. Cryptorchidism in LhrKO animals and the effect of testosterone-replacement therapy. Hum Reprod. 2006 Apr);21(4):936–942. 10.1093/humrep/dei433 16361283

[38] Radovick S, Wray S, Lee E, Nicols DK, Nakayama Y, Weintraub BD, et al. Migratory arrest of gonadotropin-releasing hormone neurons in transgenic mice. Proc Natl Acad Sci USA. 1991 Apr 15;88(8):3402–3406. 10.1073/pnas.88.8.3402 2014260 PMC51455

[39] Hadziselimovic F, Girard J. Pathogenesis of Cryptorchidism. Horm Res Paediatr. 1977;8(2):76–83. 10.1159/000178783 20399

[40] Hadziselimovic F, Girard J, Herzog B. Die Bedeutung des Nebenhodens für den Descensus testiculorum. Helv Paediatr Acta 45. 1980;(34):34.

[41] Grocock CA, Charlton HM, Pike MC. Role of the fetal pituitary in cryptorchidism induced by exogenous maternal oestrogen during pregnancy in mice. J Reprod Fertil. 1988 May);83(1):295–300. 10.1530/jrf.0.0830295 2899644

[42] Hadžiselimović F, Herzog B, Krušlin E. Estrogen-Induced Cryptorchidism in Animals. In: Hafez ESE, editor. Cryptorchidism: Management and Implications. New York: Springer Verlag; 1983. p. 25.

[43] Nef S, Parada LF. Cryptorchidism in mice mutant for Insl3. Nat Genet. 1999 Jul);22(3):295–299. 10.1038/10364 10391220

[44] Zimmermann S, Steding G, Emmen JMA, Brinkmann AO, Nayernia K, Holstein AF, et al. Targeted Disruption of the Insl3 Gene Causes Bilateral Cryptorchidism. Mol Endocrinol. 1999 May 1;13(5):681–691. 10319319 10.1210/mend.13.5.0272

[45] Emmen JM, McLuskey A, Adham IM, Engel W, Grootegoed JA, Brinkmann AO. Hormonal control of gubernaculum development during testis descent: gubernaculum outgrowth in vitro requires both insulin-like factor and androgen. Endocrinology. 2000 Dec);141(12):4720–4727. 10.1210/endo.141.12.7830 11108287

[46] Kubota Y, Nef S, Farmer PJ, Temelcos C, Parada LF, Hutson JM. Leydig insulin-like hormone, gubernacular development and testicular descent. J Urol. 2001 May 1;165(5):1673–1675. 10.1016/S0022-5347(05)66389-6 11342953

[47] Ferlin A, Simonato M, Bartoloni L, Rizzo G, Bettella A, Dottorini T, et al. The INSL3-LGR8/GREAT ligand-receptor pair in human cryptorchidism. J Clin Endocrinol Metab. 2003 Sep);88(9):4273–4279. 10.1210/jc.2003-030359 12970298

[48] Foresta C, Ferlin A. Role of INSL3 and LGR8 in cryptorchidism and testicular functions. Reprod Biomed Online. 2004 Sep);9(3):294–298. 10.1016/s1472-6483(10)62144-x 15353080

[49] Bogatcheva NV, Ferlin A, Feng S, Truong A, Gianesello L, Foresta C, et al. T222P mutation of the insulin-like 3 hormone receptor LGR8 is associated with testicular maldescent and hinders receptor expression on the cell surface membrane. Am J Physiol Endocrinol Metab. 2007 Jan;292(1):E138-E144. 16926383 10.1152/ajpendo.00228.2006

[50] El Houate B, Rouba H, Sibai H, Barakat A, Chafik A, Chadli EB, et al. Novel mutations involving the INSL3 gene associated with cryptorchidism. J Urol. 2007 May);177(5):1947–1951. 10.1016/j.juro.2007.01.002 17437853

[51] Canto P, Escudero I, Söderlund D, Nishimura E, Carranza-Lira S, Gutierrez J, et al. A novel mutation of the insulin-like 3 gene in patients with cryptorchidism. J Hum Genet. 2003;48(2):86–90. 10.1007/s100380300012 12601553

[52] Tomboc M, Lee PA, Mitwally MF, Schneck FX, Bellinger M, Witchel SF. Insulin-like 3/relaxin-like factor gene mutations are associated with cryptorchidism. The Journal of Clinical Endocrinology and Metabolism. 2000 Nov);85(11):4013–4018. 10.1210/jcem.85.11.6935 11095425

[53] Gorlov IP, Kamat A, Bogatcheva NV, Jones E, Lamb DJ, Truong A, et al. Mutations of the GREAT gene cause cryptorchidism. Hum Mol Genet. 2002 Sep 15;11(19):2309–2318. 10.1093/hmg/11.19.2309 12217959

[54] Baker LA, Nef S, Nguyen MT, Stapleton R, Nordenskjold A, Pohl H, et al. The insulin-3 gene: lack of a genetic basis for human cryptorchidism. J Urol. 2002 Jun);167(6):2534–2537. 11992081

[55] Koskimies P, Virtanen H, Lindström M, Kaleva M, Poutanen M, Huhtaniemi I, et al. A Common Polymorphism in the Human Relaxin-Like Factor (RLF) Gene: No Relationship with Cryptorchidism. Pediatr Res. 2000 Apr);47(4):538–541. 10.1203/00006450-200004000-00020 10759163

[56] Lim HN, Raipert-de Meyts E, Skakkebaek NE, Hawkins JR, Hughes IA. Genetic analysis of the INSL3 gene in patients with maldescent of the testis. Eur J Endocrinol. 2001 Feb);144(2):129–137. 10.1530/eje.0.1440129 11182749

[57] El Houate B, Rouba H, Imken L, Sibai H, Chafik A, Boulouiz R, et al. No association between T222P/LGR8 mutation and cryptorchidism in the Moroccan population. Horm Res. 2008;70(4):236–239. 10.1159/000151596 18772597

[58] Nuti F, Marinari E, Erdei E, El-Hamshari M, Echavarria MG, Ars E, et al. The leucine-rich repeat-containing G protein-coupled receptor 8 gene T222P mutation does not cause cryptorchidism. J Clin Endocrinol Metab. 2008 Mar);93(3):1072–1076. 10.1210/jc.2007-1993 18073304

[59] Josso N, Belville C, di Clemente N, Picard JY. AMH and AMH receptor defects in persistent Müllerian duct syndrome. Hum Reprod Update. 2005 Aug);11(4):351–356. 10.1093/humupd/dmi014 15878900

[60] Hutson JM. A biphasic model for the hormonal control of testicular descent. Lancet. 1985 Aug 24;2(8452):419–421. 10.1016/s0140-6736(85)92739-4 2863447

[61] Lyet L, Vigier B, Schoot P van der. Anti-Müllerian hormone in relation to the growth and differentiation of the gubernacular primordia in mice. Reproduction. 1996 Nov 1;108(2):281–288. 10.1530/jrf.0.1080281 9038787

[62] Behringer RR, Finegold MJ, Cate RL. Müllerian-inhibiting substance function during mammalian sexual development. Cell. 1994 Nov 4;79(3):415–425. 10.1016/0092-8674(94)90251-8 7954809

[63] Bartlett JE, Lee SMY, Mishina Y, Behringer RR, Yang N, Wolf J, et al. Gubernacular development in Müllerian inhibiting substance receptor-deficient mice. BJU Int. 2002;89(1):113–118. 10.1046/j.1464-410X.2002.02530.x 11849175

